# Frictional behaviour of plant proteins in soft contacts: unveiling nanoscale mechanisms†‡

**DOI:** 10.1039/d2na00696k

**Published:** 2022-12-26

**Authors:** Evangelos Liamas, Simon D. Connell, Anwesha Sarkar

**Affiliations:** a Food Colloids and Bioprocessing Group, School of Food Science and Nutrition, University of Leeds UK A.Sarkar@leeds.ac.uk; b Molecular and Nanoscale Physics Group, School of Physics and Astronomy, University of Leeds UK

## Abstract

Despite the significance of nanotribology in the design of functional biomaterials, little is known about nanoscale friction in the presence of protein-coated soft contact surfaces. Here, we report a detailed investigation of frictional behaviour of sustainable plant proteins at the nanoscale for the first time, using deformable bio-relevant surfaces that achieve biologically relevant contact pressures. A combination of atomic force microscopy, quartz crystal microbalance with dissipation monitoring, and friction force microscopy with soft polydimethylsiloxane (PDMS, 150 kPa) surfaces was employed to elucidate the frictional properties of model plant proteins, *i.e.* lupine, pea, and potato proteins at the nanoscale while systematically varying the pH and ionic strength. Interactions of these plant proteins with purified mucins were also probed. We provide the much-needed direct experimental evidence that the main factor dictating the frictional properties of plant proteins is their affinity towards the surface, followed by the degree of protein film hydration. Proteins with high surface affinity, such as pea and potato protein, have better lubricating performance than lupine at the nanoscale. Other minor factors that drive lubrication are surface interactions between sliding bodies, especially at low load, whilst jamming of the contact area caused by larger protein aggregates increases friction. Novel findings reveal that interactions between plant proteins and mucins lead to superior lubricating properties, by combining high surface affinity from the plant proteins and high hydration by mucins. We anticipate that fundamental understanding gained from this work will set the stage for the design of a plethora of sustainable biomaterials and food with optimum nanolubrication performance.

## Abstract 2

Nanoscale friction of protein involves a subtle interplay between adhesion to the surface, protein hydration, load and affinity towards the surface.

## Graphical abstract

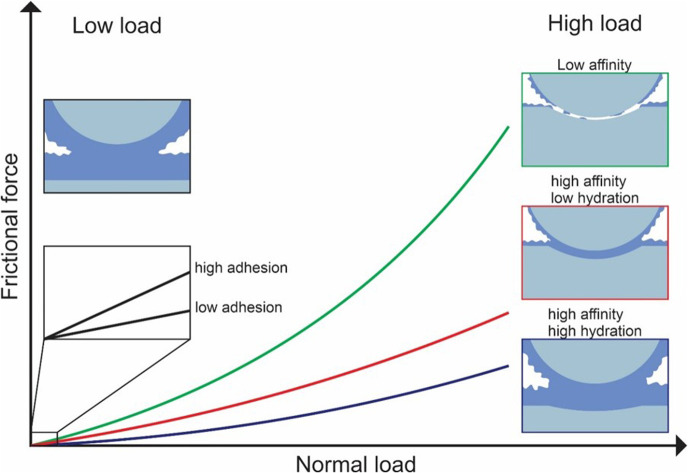

## 1. Introduction

Proteins possessing an array of genetically encoded structures and unique mechanical features have been widely exploited to create a rich palette of functional biomaterials with tailored properties. Due to a recent focus on attaining environmental sustainability and net zero, there is an increasing need to shift towards the utilization of alternative and environmentally friendly protein sources for designing protein-based biomaterials. Consequently, there is an upsurge in research efforts on understanding the structure and properties of plant proteins across multiple length scales for their application in food, feed, biomedical, biotechnological and allied soft matter sectors. Often plant proteins are associated with astringency and dryness, an unpleasant sensation during oral processing. This causes poor outcomes when used in food, oral care applications and oral medicines, particularly hindering large scale acceptance for the transition from animal proteins. Such astringency has been often attributed to the lubrication failure of salivary proteins, though the exact mechanism remains elusive.^1–3^

Tribology, the science of friction, lubrication and wear has emerged as a mechanical tool to quantify such perceived astringency where higher friction coefficients have been correlated with astringency perception.^4–8^ While there has been an increasing research effort into understanding these phenomena, they have exclusively taken place at the macroscale.^9^ The tribological behaviour of plant proteins at the nanoscale, which can shed light on the fundamental mechanism behind astringency, remains unexplored. Nanotribology is the field that studies frictional energy dissipation at the nanoscale, where adhesion and contact area can affect the tribological properties of a system more significantly, as compared to a macroscale system. Friction force microscopy (FFM), which is based on atomic force microscopy (AFM), is one of the most versatile instruments being used to study friction at the nanoscale.^10^ One of its advantages is that bespoke colloidal probes can be utilized with tailored mechanical and chemical properties similar to those found in biological tissue. Consequently, FFM has provided some valuable insight into the role of proteins in lubrication, with most of this research focusing on the lubricating properties of synovial fluid and its components.^11–15^ Protein lubrication is not only affected by the type (hydrophobicity, structure, charge) and adsorption properties of the protein, but it is also dependent on the deformability of the surfaces that are used as tribopairs. For instance, while the friction coefficient between bovine submaxillary mucin (BSM)-coated poly(methyl methacrylate) (PMMA) bodies is 0.7,^16^ it is reduced to 0.3 on BSM-coated polydimethylsiloxane (PDMS) surface as measured by a sharp silicon nitride AFM tip, and it is increased when the AFM tip is hydrophobised due to increased adhesion.^17^

Our recent study used a combination of hard (borosilicate glass) and soft (PDMS) colloidal probes to measure friction on animal protein-coated PDMS surfaces with varying modulus.^18^ It was demonstrated that the lubricating properties of these proteins were affected both by the hydrophilicity and stiffness of the colloidal probe, as well as the elastic modulus of the underlying surface. It was concluded that on such protein-coated surfaces, reduction of elastic modulus leads to reduced friction, as a result of greater load distribution with a correlated reduction in maximum pressure and lower protein ploughing from the interface. A recent development in macroscale studies is the systematic shift to design 3D printed bio-relevant surfaces replicating the tongue surface, with a particular focus on biological papillae-like roughness and wettability.^19^ However, some major drawbacks of macroscopic experiments, such as the relatively high load conditions, and the low sensitivity to the changes occurring on the lubricating film, obscure interpretation of the lubricating behaviour in terms of the adsorption and desorption of the proteins on surfaces.

Nanotribology addresses this challenge by using extremely low (∼nN) normal forces along with controlled modulus soft (∼kPa) deformable colloidal probes and surfaces, giving access to surface contact pressures orders of magnitude lower.^10^ It also enables to access and focus on a regime such as the tongue papillae (∼μm), to better understand its role on the overall frictional dissipation of the oral cavity. Therefore, FFM on soft surfaces enables tribological measurements into a realistic physiological regime, equating to surface contact pressures of <50 kPa at the lower end of the force range covered in this study (specifically at <10 nN normal force), a range experienced in the oral cavity, where tongue pressures of 40–80 kPa are typical in healthy adults.^20–22^ Furthermore, this tribological study with plant proteins gives an unprecedented understanding of the dynamics when a plant protein moiety encounters a single papilla in a tongue rather than the entire tongue. Understanding the frictional behaviour of plant proteins and their interaction with mucin-coated surfaces, a representative of many biological surfaces, and in particular oral mucosa, will enable optimizing plant proteins effectively to be used for biomaterial design intended for the oral administration route.

Lubrication is often directly correlated to protein adsorption.^8^ Quartz crystal microbalance with dissipation (QCM-D) allows real-time monitoring of protein adsorption on a range of surfaces, together with important information regarding the viscoelastic properties of the adsorbed film.^23^ QCM-D has been used in the past to understand the lubricating properties of salivary components.^23^

Mucins are physiologically relevant proteins coating the inner epithelium providing lubricity and protection against wear. Mucins such as MUC1, MUC4, and MUC16 reduced the friction coefficient (*μ*) from ∼0.20 to ∼0.08, particularly in the presence of a purified gel-forming secretory mucin, MUC2.^24^ In another study, it was found that salivary mucin on its own is not a good lubricant, but when combined with smaller proteins such as lactoferrin, a synergistic effect is created leading to superior lubricating properties.^25^ In another study, it was found that while pea protein adsorbs at higher amounts than whey protein, it had inferior lubrication performance.^26^ Similar results were reported with a wider selection of plant proteins such as pea, potato and lupine,^27^ with inferior lubricity attributed to the jamming of the contact zone, which correlated with the increased adsorbed mass.

Besides the type of surface used, two main factors that can affect the adsorption of proteins, and thus lubrication, are pH and ionic strength. An increase in ionic strength reduces the Debye screening length and as a result, it decreases both electrostatic repulsion between like-charged surfaces (increases adsorption) as well as the electrostatic attraction between oppositely charged surfaces (decreases adsorption), as was shown in a range of proteins and surfaces.^28–30^ Similarly, a change in pH will affect the net charge of a protein molecule.^31^ Interestingly, it has been shown that adsorption on uncharged surfaces is slow and non-specific which suggests the impact of pH and ionic strength on protein adsorption is not often straightforward.^32^

Therefore, in this study, we uniquely combined FFM, AFM and QCM-D to understand the lubrication performance of plant proteins at the nanoscale, and demonstrated how they are affected by pH and ionic strength, using bio-relevant soft surfaces. Lupine, pea, and potato proteins were used as model plant proteins, which are composed mainly of conglutin, legumin, and patatin, respectively. However, they also contain a range of other proteins, such as albumin, vicilin and convicilin, as well as smaller molecules such as protease inhibitors and enzymes. Although mucin alone cannot replicate the this complex salivary pellicle completely,^25,33^ it can represent a wide range of oral and other biological tissues. Novel findings reveal that the frictional properties of proteins are affected by changes in both pH and ionic strength, which are driven by factors such as total adsorbed hydrated mass, protein affinity towards the underlying substrate, degree of protein hydration, surface roughness, as well as the presence of mucins. To our knowledge, this is the first systematic investigation of the nanotribological behaviour of plant proteins in soft bio-relevant contacts and may lead to innovative strategies for the rational design of sustainable food and biomaterials.

## 2. Results and discussion

### 2.1. Adsorption and viscoelastic properties of plant protein films

In order to study the lubricating properties of plant proteins, it is important to understand the physical properties of the films they generate in real-time on bio-relevant surfaces, and how they adsorb and desorb. Polydimethylsiloxane (PDMS) is a conventional elastomer that is widely used to replicate the surfaces found in the oral cavity due to its relatively low modulus compared to steel and glass counterparts.^34^ Still, PDMS has a modulus of around 2 MPa, which is in the upper range of those found in biological tissues (a few kPa to a few MPa).^35^ It is now experimentally evidenced by our group that elastic modulus can significantly alter the lubricating properties of protein-coated surfaces.^18^ To this end, PDMS surfaces with a biologically relevant modulus of 150 kPa were employed as surfaces to test the lubrication and adsorption performance of lupine (Lup), pea (Pea), and potato (Pot) proteins,^18^ using contact pressures <50 kPa, in the physiological regime.^20–22^ Different food-relevant pH and ionic strength conditions that can occur orally were chosen *i.e.* pH 3.0 and 10 mM NaCl, pH 7.0 and 10 mM NaCl, and pH 7.0 and 50 mM NaCl, a minimal sample set to test the effect of both pH and ionic strength as electrostatic interactions are expected to play a pivotal role in the formation and stability of charged protein films in the oral cavity. These conditions will be referred to as 310, 710, and 750, respectively. In addition to bare PDMS surfaces, mucin (BSM)-coated PDMS surfaces were also used to serve as proxies for salivary pellicles coating oral surfaces.

**Fig. 1:**
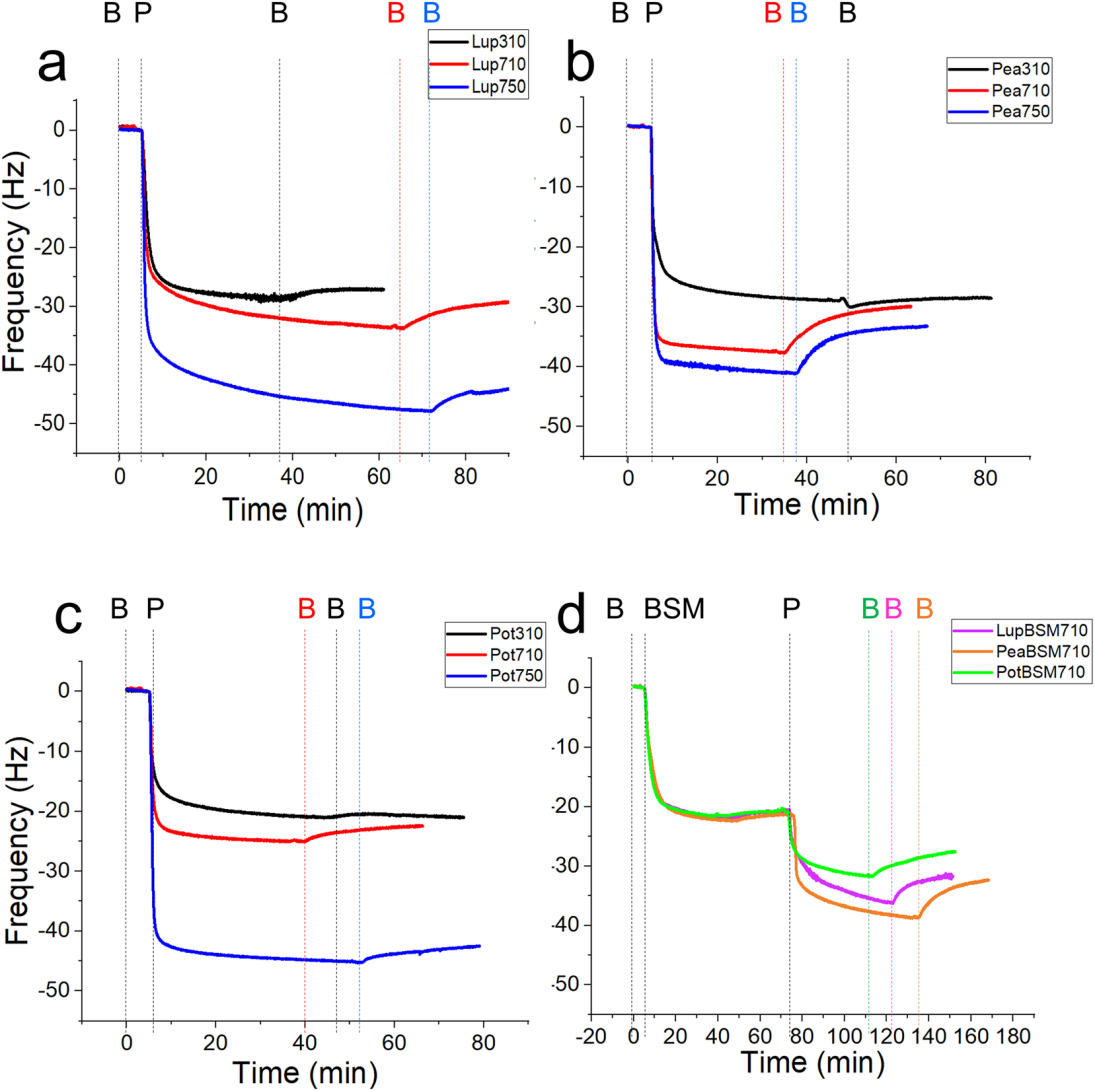
Mean frequency shift (5th overtone) of lupine (a), pea (b) and potato (c) protein films (*n* = 3) adsorbed on bare polydimethylsiloxane (PDMS) coated sensors, acquired by quartz crystal microbalance with dissipation monitoring (QCM-D). At bare PDMS surfaces, measurements were taken in presence of 1 mg mL^−1^ protein dissolved at three different pH and ionic concentration combinations: 10 mM NaCl at pH 3.0 (Lup310, Pea310, Pot310), 10 mM NaCl at pH 7.0 (Lup710, Pea710, Pot710), and 50 mM NaCl at pH 7.0 (Lup750, Pea750, Pot750). Mean frequency shift (5th overtone) of lupine, pea and potato protein films (*n* = 3) adsorbed on BSM-coated surfaces (d) were performed in presence of 1 mg mL^−1^ protein dissolved in 10 mM NaCl at pH 7.0 (LupBSM710, PeaBSM710, PotBSM710). Steps B, P, and BSM refer to buffer rinsing, protein addition, and mucin coating, respectively.

**Fig. 2:**
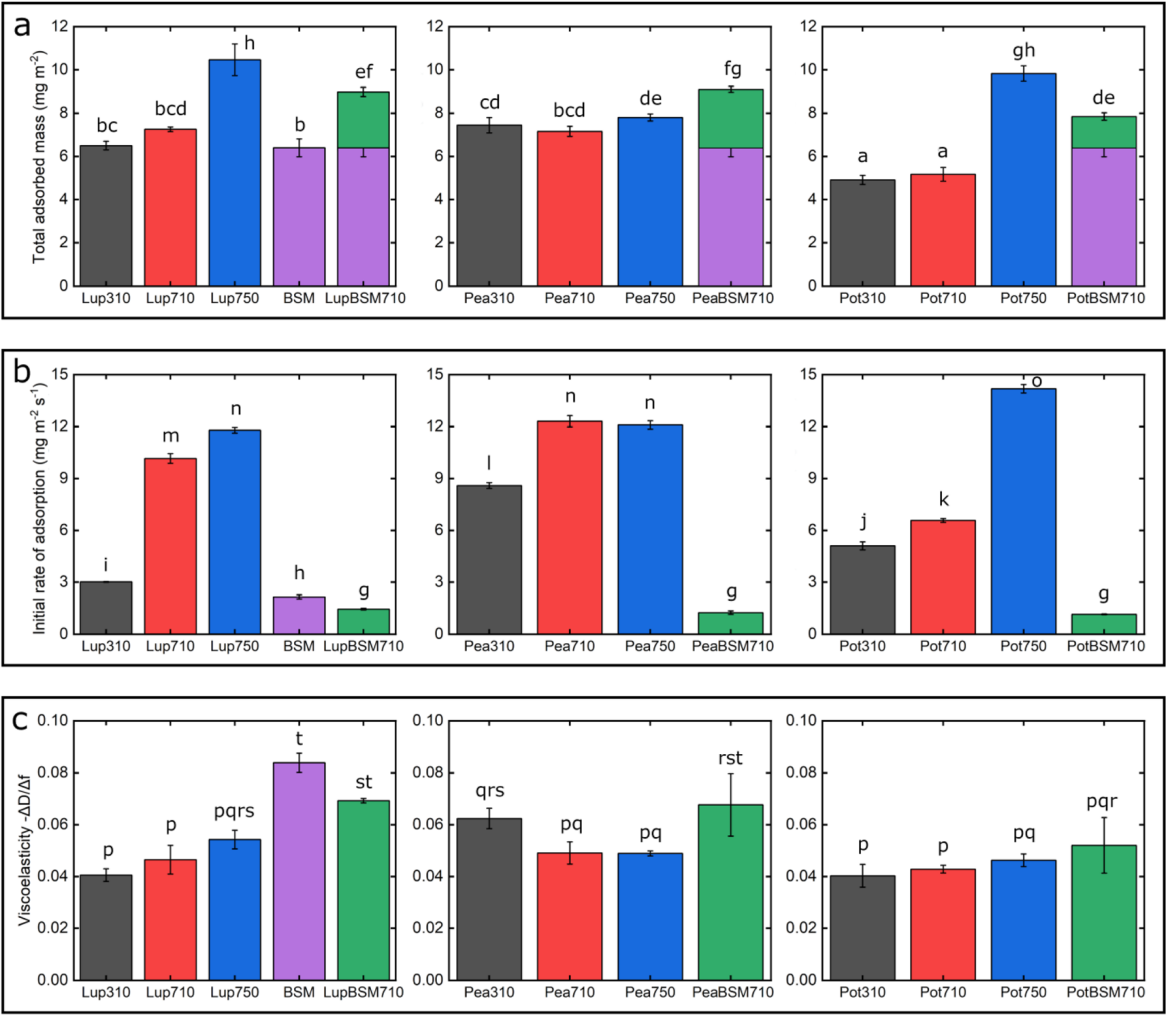
Adsorbed hydrated mass (a), adsorption kinetics (b), and viscoelastic properties −Δ*D*/Δ*f* (c) of lupine, pea, and potato protein films (*n* = 3, mean ± SD) adsorbed on polydimethylsiloxane (PDMS) coated sensors, acquired by quartz crystal microbalance with dissipation monitoring (QCM-D). At bare PDMS surfaces measurements were taken in presence of 1 mg mL^−1^ protein dissolved at three different pH and ionic concentration combinations: 10 mM NaCl at pH 3.0 (Lup310, Pea310, Pot310), 10 mM NaCl at pH 7.0 (Lup710, Pea710, Pot710), and 50 mM NaCl at pH 7.0 (Lup750, Pea750, Pot750). Measurements on BSM-coated surfaces were performed in presence of 1 mg mL^−1^ protein dissolved in 10 mM NaCl at pH 7.0 (LupBSM710, PeaBSM710, PotBSM710). The purple bar shows BSM coating and green bar shows protein adsorption on BSM-coated surfaces. Samples with the same letter do not differ significantly (*p* > 0.05) according to Tukey's test.

First, we report the trend of real-time adsorption behaviour on bare PDMS sensors (see Fig. 1a–d for frequency shifts, Fig. S1a–d‡ for corresponding dissipation shifts) and discuss the data fitted with the Voigt viscoelastic model (Fig. 2) followed by any deviations observed on the BSM-coated PDMS sensors.^36^ After injection of proteins (step P), an immediate large reduction in frequency indicates that proteins, irrespective of their types, pH and ionic conditions, adsorbed on the bare PDMS surfaces, reaching a critical coverage and stabilizing at −20 to −48 Hz before they slightly desorb upon rinsing with clean buffer (step B, Fig. 1a–d). The higher energy dissipation values during the formation of protein films (0.8–2.3 ppm) (Fig. S1a–d‡) indicate that plant protein films are not rigid, but rather viscoelastic.^27^

One common trend is that the presence of 50 mM NaCl always increases the degree of adsorption indicated by a higher frequency reduction (Fig. 1) and increased film viscosity (Fig. S1‡) irrespective of the protein type, highlighting that charge screening substantially enables a close approach of the plant proteins closer to the PDMS surface and the formation of a thicker protein film. This response is statistically valid in the case of Lup750 and Pot750 (Fig. 2a) where the total adsorbed mass is higher than their counterparts at lower ionic strengths (*p* < 0.05). Interestingly, this corroborates fully with the adhesive forces between a PDMS probe and a PDMS surface in the presence of plant proteins (Fig. S2‡), as calculated with force–distance curves (Fig. S3‡) by performing force spectroscopy, where the presence of 50 mM NaCl substantially increases adhesion as compared to the lower salt concentration samples at same pH (*p* < 0.05).

Of note, the affinity towards the surface, indicated by the initial rate of protein adsorption (Fig. 2b) and time required to reach stabilization, as well as the absolute magnitude of frequency shift (Fig. 1) varies significantly depending on the type of proteins and conditions, even if the viscoelasticity and consequently protein hydration remains more or less constant (Fig. 2c).

For instance, Lup310 stabilizes in almost half the time as required by Lup710/Lup750 (>60 min) (Fig. 1a) with the Lup310 forming a rather rigid film (dissipation shift ∼1 ppm) (Fig. S1b‡), despite the initial adsorption rate of Lup310 being three to four time slower, highlighting the importance of pH to the adsorption rate and final film structure (Fig. 1a). It is noteworthy that the isoelectric point (pI) of lupine is close to 4.0 (Table S1‡),^37^ and hence Lup310 being in the vicinity of its pI might lead to protein–protein aggregation hindering their adsorption and consequent adhesion to PDMS surfaces. Not surprisingly, the proteins tested showed significant differences in their affinity (Fig. 2b) depending on the protein type (*p* < 0.05) attributable to their hydrophobic residue composition and amino acid sequences. However, a shift in pH from acidic to neutral always increased the initial rate of adsorption of the proteins to the bare PDMS surfaces (*p* < 0.05) (Fig. 2b), which also increased adhesive forces between the two PDMS surfaces except for Pot (Fig. S2‡).

One striking feature specific to Pea was that the extent of desorption upon buffer rinsing was much higher for Pea710 and Pea750 (>+8 Hz frequency shift) as compared to corresponding Lup (Fig. 1a) and Pot (Fig. 1c) counterparts. Such desorption behaviour was specific to pH 7.0, but not at pH 3.0 where Pea is cationic, indicating that the Pea at pH 7.0 was rather loosely bound to PDMS surface in the first place, as well as a weak protein–protein interaction. Unlike Lup and Pot, Pea also did not show such striking difference in either frequency shift (Fig. 1b), energy dissipation (Fig. S1b‡) or initial rate of adsorption (Fig. 2b) as a function of ionic strength. Out of all the proteins, Pot protein demonstrated rapid stabilization within the first 10 min of injection (Fig. 1c) showing very similar behaviour to Lup (Fig. 1a) in terms of ionic strength effects reinforcing adsorption (Fig. 1b). Nevertheless, the change in pH (Pot310 *vs.* Pot710) did not appear to affect the energy dissipation (Fig. S1c‡), as previously shown in the case of Lup counterparts (Fig. S1a‡). In summary, neutral pH and higher ionic strength improve the affinity of plant proteins for bare PDMS, but the absolute mass and adsorption rate are dependent on protein type, whilst having little influence on the viscoelasticity of the films formed.

Having discussed the adsorption behaviour on bare PDMS surfaces, it appears that on surfaces pre-coated with BSM, the difference between the protein types diminishes in both adsorption (Fig. 1d) and dissipation (Fig. S1d‡). Adsorption of BSM onto PDMS occurs rather slowly (Fig. 2b) as compared to plant proteins, indicating lower affinity of BSM towards the PDMS surface, while it forms a highly viscous/hydrated film (Fig. 2c). This is expected owing to the large macromolecular conformation of BSM that hinders its ability to reach the surface quickly, and presence of several hydroxyl groups in the glycan chains of BSM which increases protein hydration and energy dissipation of BSM films (Fig. S1d‡). Adsorption of Lup710, Pea710, and Pot710 on BSM occurs very slowly as opposed to bare hydrophobic PDMS surfaces (Fig. 2b), indicating a small affinity of the plant proteins towards BSM-coated surfaces. This is further evidenced by a significant reduction of adhesive forces of plant proteins in the presence of BSM (*p* < 0.05) (Fig. S2‡) arising from the BSM-protein repulsive forces, as plant proteins and BSM are all negatively charged at pH 7.0.

To obtain more information about protein film coverage, atomic force microscopy (AFM) was used to image pea, lupine, and potato protein films on PDMS and BSM-coated PDMS films at pH 7.0 and 10 mM NaCl. Topographic images of the protein films are shown in Fig. S4.‡ Fig. S4a‡ shows the bare PDMS surface, revealing a characteristic porous polymer network with higher roughness (25 nm peak-to-peak (p–p), 1.2 nm RMS, Table S2‡). Protein adsorption acts to progressively fill the pores in the PDMS, reducing roughness (9–17 nm p–p, 0.8–1.0 nm RMS). Adsorption of lupine protein (Lup710) on PDMS (Fig. S4c‡) results in the smallest reduction in roughness (17 nm), with some areas that appear well-coated, while other areas remain minimally coated since the characteristic porous network is still visible.

On the other hand, adsorption of pea protein (Pea710) (Fig. S4e‡) results in improved coverage as compared to Lup710 since the features of the PDMS substrate can be seen only in a few areas, and has the lowest roughness (Table S2‡) of the plant proteins as the protein completely fills and smooths the porous PDMS network. Although pea appears to have a superior coverage as compared to lupine, QCM-D shows a similar adsorbed mass, which is explained by aggregation of the lupine and potato protein (Fig. S4c‡) resulting in a similar mass but patchier coverage. Potato protein (Pot710) (Fig. S4g‡) has similar coverage to Lup710 although less and/or smaller aggregates are formed, and this is confirmed by a reduced adsorbed mass (Fig. 2a).

Coating the PDMS surface with BSM (Fig. S4b‡) results in a more uniform and smoother protein film, coating the underlying PDMS surface, which is in line with QCM-D data (Fig. 1d). Adsorption of lupine protein (Fig. S4d‡) and pea protein (Fig. S4f‡) on BSM-coated PDMS results in coverages that are far superior as compared to adsorption of lupine and pea on bare PDMS. Although the adsorption of potato protein on BSM (Fig. S4h‡) improves coverage as compared to the adsorption of potato alone on PDMS, it was far less complete as compared to those of lupine and pea on BSM. This is also supported by the reduced surface roughness of plant proteins adsorbed on BSM as compared to adsorption on PDMS (Table S2‡), while QCM-D also revealed a significantly increased hydrated mass of the BSM + plant protein films (*p* < 0.05) (Fig. 2a).

Lastly, it appears that lupine and pea proteins have higher affinity with BSM, resulting in a compact protein film that coats uniformly the PDMS surface, while the affinity of potato towards BSM is rather smaller, which is also supported by QCM-D data (Fig. 1b). Nevertheless, irrespective of plant protein type, the presence of BSM allows the formation of a more compact and continuous film on the underlying PDMS surface (Fig. S4d, f and h‡) as compared to a rather discontinuous film in its absence. Such differences in surface coverage might have some impact on the tribological performance of the proteins, which is discussed in the following section.

### 2.2. Effect of pH and ionic strength on the lubricating properties of plant proteins at the nanoscale

**Fig. 3:**
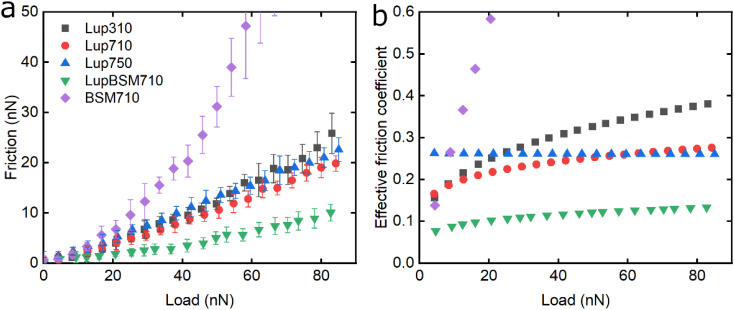
Evolution of friction force *versus* load (*n* = 3, mean ± SD) (a) and effective friction coefficient *versus* load (b) of a polydimethylsiloxane (PDMS) colloidal probe (*E* = 2 MPa) sliding over bare and BSM-coated PDMS substrates (*E* = 150 kPa), acquired by friction force microscopy. At bare PDMS surfaces, measurements were performed in presence of 1 mg mL^−1^ lupine protein dissolved at three different pH and ionic concentration combinations: 10 mM NaCl at pH 3.0 (Lup310), 10 mM NaCl at pH 7.0 (Lup710), and 50 mM NaCl at pH 7.0 (Lup750). Measurements on BSM-coated surfaces (1 mg mL^−1^ BSM, BSM710) were performed in presence of 1 mg mL^−1^ lupine protein dissolved in 10 mM NaCl at pH 7.0 (LupBSM710).

Following the characterisation of plant protein films, friction force microscopy was used to study the friction between a PDMS colloidal probe (Fig. S5‡) and soft PDMS surfaces in the presence of plant proteins. In all the following discussion, BSM is shown as a control (Fig. 3, 5 and 6). BSM exhibits strikingly high friction, the friction increases exponentially with normal load, experiencing a high degree of dehydration and/or protein removal, while the friction coefficient is approximately 0.2 at 5 nN load and increases up to 2.2 at 80 nN load. The poor lubricating properties of BSM are a result of its very low affinity towards the PDMS surface, as shown in Fig. 2b. Consequently, although it forms a highly viscous film (Fig. 2c), the protein film is easily removed from the surface, which increases friction with increasing load. Generally, the more hydrated a protein film the lower the friction due to improved lubrication, provided the film remains at the surface during exposure to tribo-stress. However, if the macromolecule is not strongly adsorbed on the surface, then it can be easily removed upon sliding. It is worth noting that all plant proteins on their own had lower friction coefficients than BSM, particularly at higher loads (Fig. 3, 5 and 6).

Moving onto the frictional dissipation of individual plant proteins, friction increases as a function of normal load (Fig. 3a) in the presence of lupine, with pH and ionic strength not having a major impact on friction force for the range studied here. For a multi-asperity nanoscale contact with negligible adhesion, it would be expected that friction force would increase linearly with the load. However, it can be observed that friction force increased exponentially with the load. This is a behaviour that has been observed recently, and it is caused by the removal and dehydration of the adsorbed protein film.^18^ In order to calculate and plot an effective friction coefficient (*μ*_eff_) from the slope (Fig. 3b), the data were fitted using an allometric equation (*y* = *a* + b*x^c^, *a* = 0), and the gradient was then calculated at each friction-load point. Factor *α* was set to zero to ensure a good fit since adhesion on these systems is very low (Fig. S2‡) and, thus, friction at zero load is approximately zero (as shown to be the case in Fig. 3, 5 and 6).

Lup310 and Lup710 exhibit similar *μ*_eff_ at low loads, at approximately 0.15. However, at high loads, *μ*_eff_ is lower for Lup710 (0.30) as compared to 0.37 for Lup310, the latter exhibiting the highest increase in *μ*_eff_ (Fig. 3b), which might be associated with load-induced higher dehydration and/or protein removal in case of Lup310. Lupine has net negative charge at pH 7.0 and a net positive charge at pH 3.0.^38^ Since PDMS is a hydrophobic surface (non-polar), electrostatic interactions are not expected to play a role in the adsorption of proteins. However, the lower affinity of lupine molecules towards the PDMS surface at pH 3.0 compared to pH 7.0 due to minor changes in the molecular structure of lupine, as evidenced by QCM-D dissipation (Fig. 2a) data, and by adhesion data (Fig. S2‡), most likely leads to increased protein removal and consequently higher friction coefficient, especially at high loads (Fig. 3b).

Considering that the usual increase in *μ*_eff_ between low and high loads is often explained by dehydration and/or protein film removal,^18^ such phenomenon does not seem to hold true for Lup750, revealing an almost constant *μ*_eff_ at approximately 0.26 independent of load. This indicates that Lup750 shows the highest resistance to dehydration and/or protein removal with load independency for the range studied here, followed by Lup710 and lastly by Lup310. It is well established that increased salt concentration leads to improved protein hydration; a larger number of ions are bound on charged regions of the protein that in turn increases the number of water molecules bound on them.^39–42^ Consequently, the increased ionic concentration improves the hydration of lupine molecules, which combined with an increased protein–protein interaction reduces the dehydration and protein removal at higher loads. This is supported by QCM-D data (Fig. 1c) where Lup750 forms a more hydrated film as compared to Lup710. Although Lup750 exhibits superior lubrication performance at high loads, resisting dehydration and possibly removal, at low loads, Lup750 exhibits relatively poor lubricating properties (Fig. 3b). This is counterintuitive since Lup750 reveals a faster rate of adsorption and hydration as compared to Lup710 (Fig. 2b). AFM measurement of adhesion between the protein-coated colloidal PDMS probe and PDMS surface reveal significantly stronger probe-surface interaction for Lup750, which is 3 times stronger than Lup710, latter in turn is three times stronger than Lup310 (Fig. S2‡). Therefore, it is likely that this stronger adhesion which sets a minimum normal force is the key reason behind high friction in Lup750 at low load, and hence *μ*_eff_ is invariant with load in the force range studied. It is also possible that Lup750 creates a rather aggregated protein layer due to salt-induced aggregation, which induces roughness that affects friction. As the load increases and the protein asperities are squeezed out, it is the protein hydration and affinity that determine lubrication (Fig. 3b).

**Fig. 4:**
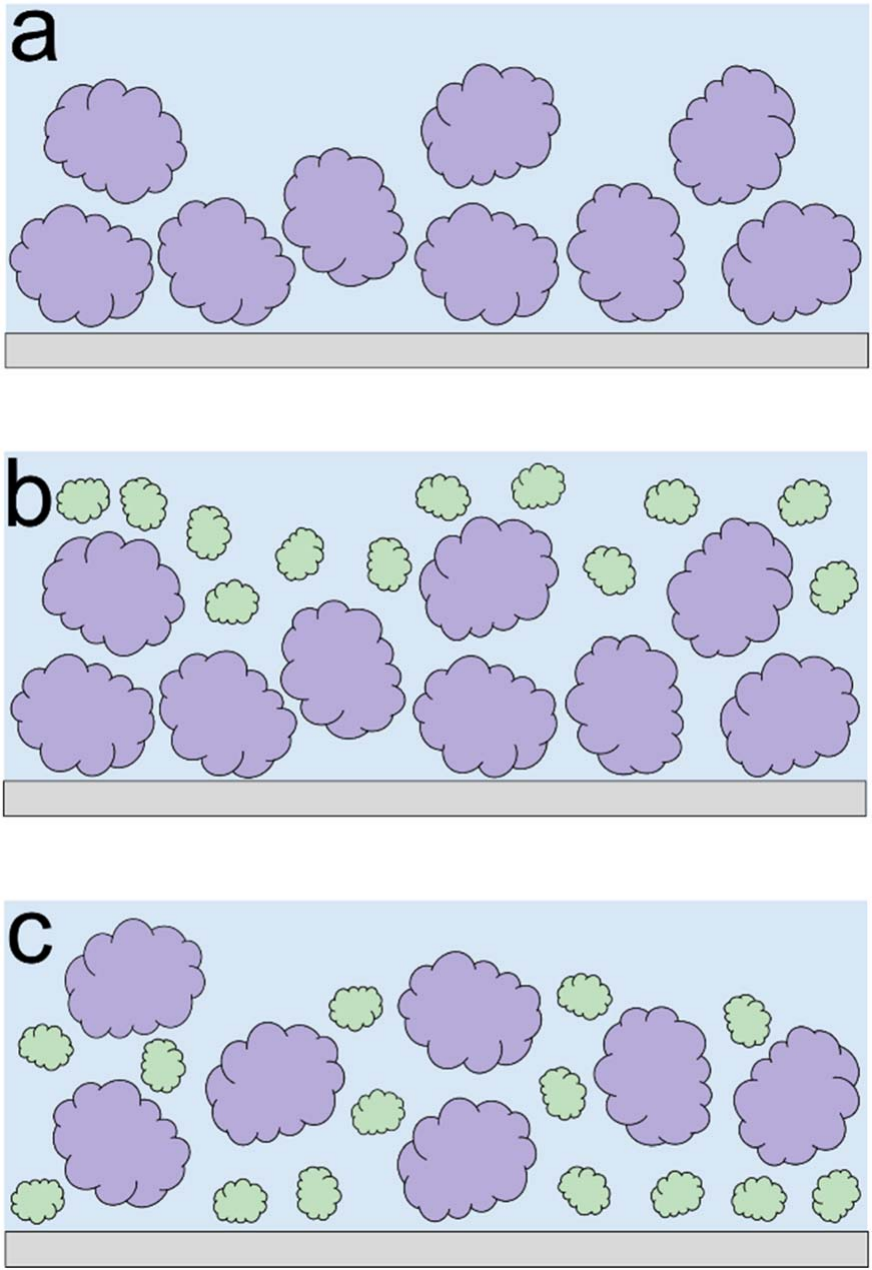
Schematic illustration protein adsorption, exhibiting the synergy between BSM and plant proteins. Initially, BSM molecules (depicted with purple colour) are adsorbed onto the PDMS surface (a). When plant proteins (depicted with green colour) are added into the system, they initially adsorb on top of the BSM molecules (b). However, over time, plant molecules tend to competitively displace BSM molecules on the PDMS surface, resulting in higher affinity of the combined mixed film (c).

Lupine on BSM-coated surfaces exhibits the lowest friction coefficient, both in low (*μ*_eff_ is 0.07 at 5 nN) and high loads (*μ*_eff_ is 0.14 at 80 nN). As discussed previously, the lubricating properties of BSM on its own are very poor, as a result of its very low affinity towards the PDMS surface (Fig. 2b). However, when BSM is combined with lupine, it forms a protein film with superior lubricating performance as compared to the lubricating performance of its constituents. BSM has an isoelectric point at pH 3.0 and, thus, an overall negative net charge at pH 7.0 (Table S1‡).^43^ Although both lupine and BSM have overall negative charges at pH 7.0, local patches on the surface of the lupine and BSM can bind the two molecules, as seen in other protein studies,^44^ creating a mixed protein film that coats uniformly the PDMS surface as was qualitatively observed in topographic images in Fig. S4d‡. Eventually, having a higher affinity towards the PDMS surface and being smaller in size, lupine molecules will competitively displace a large proportion of the BSM molecules as a result of the Vroman effect as shown schematically in Fig. 4a–c in a step-wise manner. The resulting BSM-lupine film combining the increased affinity of lupine with the superior hydration of BSM creates a protein film that is a better lubricant than both in isolation.

**Fig. 5:**
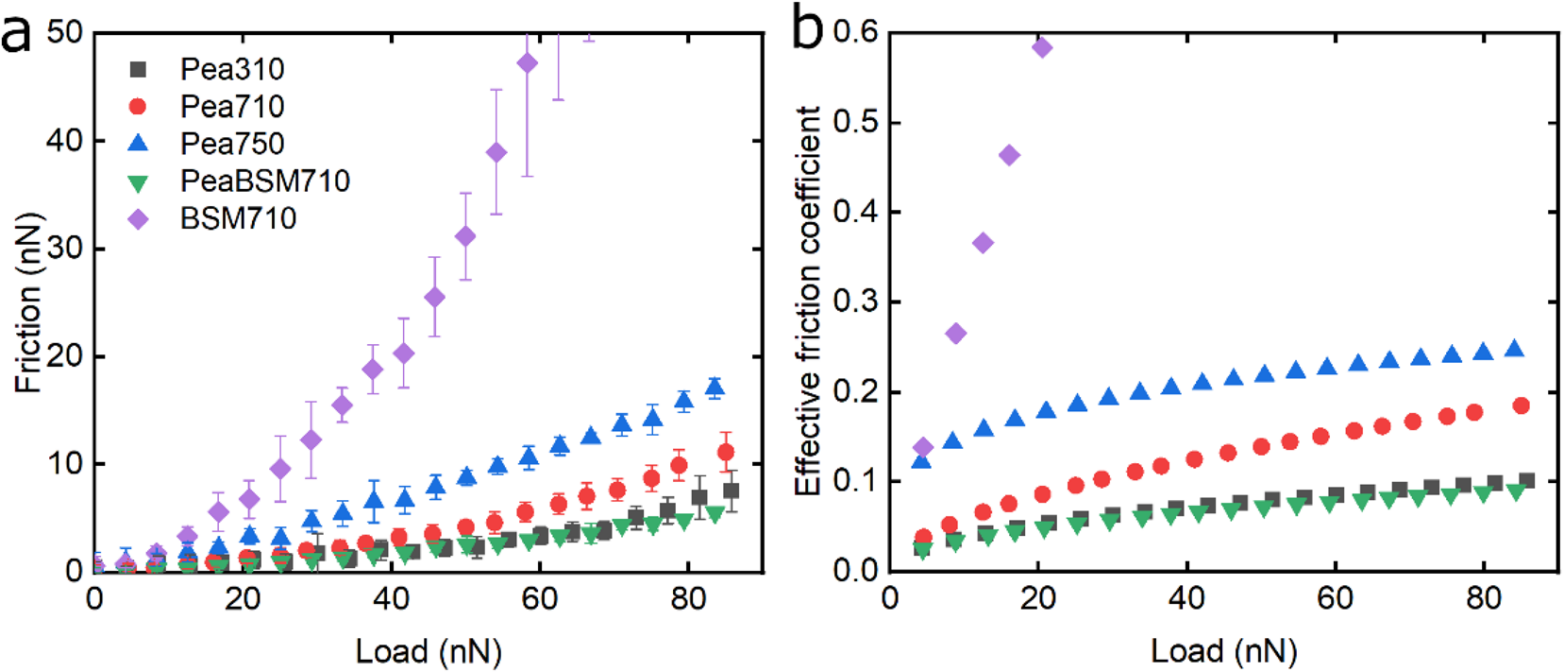
Evolution of friction force *versus* load (*n* = 3, mean ± SD) (a) and effective friction coefficient *versus* load (b) of a polydimethylsiloxane (PDMS) colloidal probe (*E* = 2 MPa) sliding over bare and BSM-coated PDMS substrates (*E* = 150 kPa), acquired by friction force microscopy. At bare PDMS surfaces measurements were performed in presence of 1 mg mL^−1^ Pea protein dissolved at three different pH and ionic concentration combinations: 10 mM NaCl at pH 3.0 (Pea310), 10 mM NaCl at pH 7.0 (Pea710), and 50 mM NaCl at pH 7.0 (Pea750). Measurements on BSM-coated surfaces (1 mg mL^−1^ BSM, BSM710) were performed in presence of 1 mg mL^−1^ pea protein dissolved in 10 mM NaCl at pH 7.0 (PeaBSM710).

The lubricating properties of pea protein are shown in Fig. 5. Unlike Lup, Pea750 *i.e.* the one containing higher salt concentration shows the highest friction, followed by Pea710, and then Pea310 with PeaBSM710 showing similar lubricating properties to that of Pea310 (Fig. 5a).

Although QCM-D revealed similar degrees of affinity and hydration between Pea710 and Pea750 (Fig. 2b), adhesion data (Fig. S2‡) confirms a higher interaction between Pea750 and surfaces as compared to those in Pea710, which might have resulted in higher friction, especially at low loads. Comparison between Pea310 and Pea710 shows that similar to lupine, at low loads, the two protein films had similar lubricating properties. However, in contrast with lupine protein, the reduction in the pH resulted in a lower friction coefficient at high loads due to lower dehydration and/or protein removal. Pea protein has an isoelectric point at pH 4.0 (Table S1‡), thus, it has a positive net charge at pH 3.0 and a negative net charge at pH 7.0.^45^ Since Pea310 has a smaller affinity towards the PDMS surface, as confirmed by adhesion (Fig. S2‡) and kinetics (Fig. 2b), it can be deduced that the reduction in the *μ*_eff_ is not caused by reduced protein removal. In fact, the reduction in *μ*_eff_ at high loads is mainly driven by increased protein hydration as confirmed in Fig. 2c. It is likely that increased protein–protein affinity of pea molecules at pH 3.0, by having a lower net charge on their surface since they are closer to their pI point, leads to the formation of larger protein aggregates and thicker film with increased hydration capacity and, in turn, lowers friction. Pea on BSM (PeaBSM710) exhibits only slightly lower *μ*_eff_ as compared to Pea710 (Fig. 5b) unlike the difference that is apparent in LupBSM750 (Fig. 3b).

**Fig. 6:**
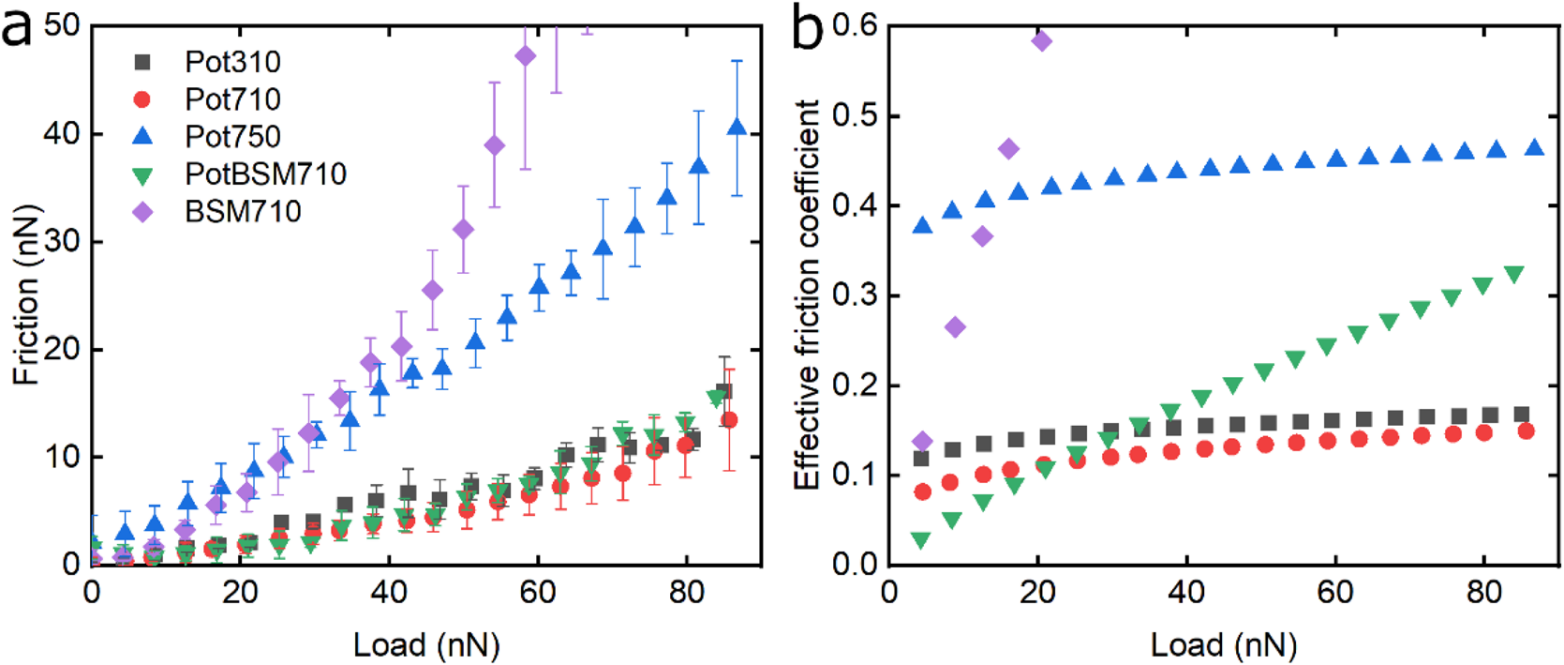
Evolution of friction force *versus* load (*n* = 3, mean ± SD) (a) and effective friction coefficient *versus* load (b) of a polydimethylsiloxane (PDMS) colloidal probe (*E* = 2 MPa) sliding over bare and BSM-coated PDMS substrates (*E* = 150 kPa), acquired by friction force microscopy. At bare PDMS surfaces measurements were performed in presence of 1 mg mL^−1^ potato protein dissolved at three different pH and ionic concentration combinations: 10 mM NaCl at pH 3.0 (Pot310), 10 mM NaCl at pH 7.0 (Pot710), and 50 mM NaCl at pH 7.0 (Pot750). Measurements on BSM-coated surfaces (1 mg mL^−1^ BSM, BSM710) were performed in presence of 1 mg mL^−1^ potato protein dissolved in 10 mM NaCl at pH 7.0 (PotBSM710).

Fig. 6 shows the lubricating properties of potato protein. Similar to pea and lupine proteins, the friction *versus* normal load curves increases exponentially (Fig. 6a). Similar to pea (Fig. 5b), Pot710 and Pot750 show that the increase of ionic strength has a major impact on the lubricating properties of potato protein, exhibiting increased *μ*_eff_ both at low and high load at higher ionic strength (Fig. 6b), unlike lupine (Fig. 4b). The increased *μ*_eff_ at low load is partially due to the increased importance of surface adhesion (Fig. S2‡). Pot750 exhibits high *μ*_eff_ even at high loads. It is possible that the increased ionic strength leads to the formation of larger aggregates that could act as particulates, jamming the contact and increasing the friction coefficient as has been shown in macroscale studies.^26,46^

Lastly, although PotBSM710 has the lowest friction at low loads, it is not resistant to dehydration and/or protein removal, which results in increased friction at higher loads as compared to Pot710. This is in contrast with the lupine and pea systems, where the underlying BSM film not only reduced friction under all conditions but also had a positive impact on the resistance of protein film to dehydration and/or protein removal. Although all three plant proteins have a similar affinity with BSM (Fig. 2b), comparing the rate of adsorption, Pot has a significantly lower affinity towards the PDMS surface as compared to Lup and Pea. Not only is it more difficult for potato protein molecules to replace BSMs, and thereby take longer time (Vroman effect, see Fig. 4a–c), the resulting film will also have a lower affinity towards the PDMS surface. Consequently, the resulting Pot/BSM film will be removed more easily from the PDMS surface, as compared to the Lup/BSM and Pea/BSM systems, especially at high loads. Additionally, potato protein appears to have impaired coverage on BSM-coated surfaces as compared to lupine and pea in the AFM topographic images (Fig. S4h‡), which might suggest that the interaction between potato protein and BSM was not promoting a compact film as in case of the other two proteins, allowing potato protein to be ploughed off at higher loads.

### 2.3. Comparison of the lubricating properties of plant proteins at the nanoscale

**Fig. 7:**
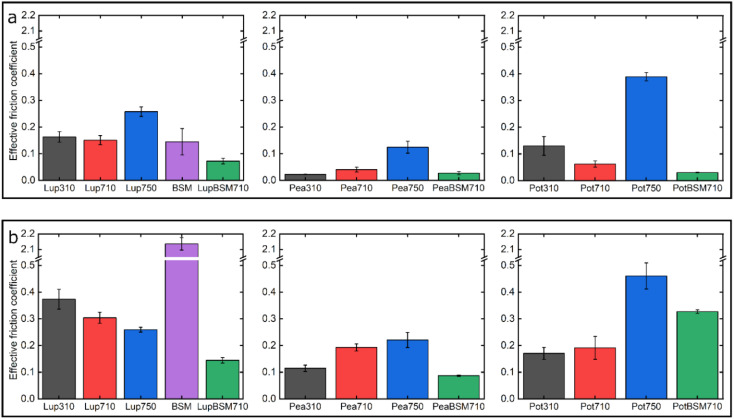
Effective friction coefficient at 5 nN low (a) and 80 nN high (b) loads of a polydimethylsiloxane (PDMS) colloidal probe (*E* = 2 Mpa) sliding over bare and BSM-coated PDMS substrates (*E* = 150 kPa), in presence of lupine, pea, and potato protein solutions, acquired by friction force microscopy. At bare PDMS surfaces measurements were taken in presence of 1 mg mL^−1^ protein dissolved at three different pH and ionic concentration combinations: 10 mM NaCl at pH 3.0 (Lup310, Pea310, Pot310), 10 mM NaCl at pH 7.0 (Lup710, Pea710, Pot710), and 50 mM NaCl at pH 7.0 (Lup750, Pea750, Pot750). Measurements on BSM-coated surfaces were performed in presence of 1 mg mL^−1^ protein dissolved in 10 mM NaCl at pH 7.0 (LupBSM710, PeaBSM710, PotBSM710).

Fig. 7 summarizes the differences in friction coefficient between lupine, pea, and potato proteins at low and high normal loads. The results demonstrate that at low loads at pH 7.0 and 10 mM NaCl, pea protein exhibits the lowest *μ*_eff_, followed by potato and then lupine protein. The superior lubricating performance of pea protein was driven by its increased affinity towards the PDMS surface (Fig. 2b), as compared to the others. Since PDMS is a hydrophobic surface, increased affinity with pea protein is due to an increased presence of hydrophobic regions on the surface of pea protein, which corroborates with previous findings.^27^ Lupine revealed similar levels of adsorbed mass (Fig. 2a) and viscoelastic properties with pea protein (Fig. 2c) but decreased affinity as was shown by the rate of adsorption (Fig. 2b). Consequently, lupine had inferior lubrication properties as compared to pea, highlighting the importance of affinity on the lubricating properties of proteins.

Potato has similar viscoelastic properties to lupine but exhibits lower affinity towards the PDMS surface which was also seen in the topographic image (Fig. S4g‡) and lower adsorbed hydrated mass (Fig. 2a). Despite this, potato has better lubricating properties than lupine at low loads, which could be attributed to the lower adhesive interactions (Fig. S2‡) which have increased importance at lower loads. These results of lubrication performance at the nanoscale in the order of pea protein > potato > lupine particularly at low loads in fact resembled the trend observed at the macroscale previously,^27^ particularly when these proteins were present at low concentrations. However, the differences between their frictional response were much more prominent at the nanoscale as compared to the macroscale and, interestingly, such nanoscale behaviour might emulate the real physiological context better owing to similar low contact pressures and reduction in protein removal.

Interestingly, when BSM was combined with lupine, pea, and potato, friction was reduced significantly in all cases. More specifically, pea and potato proteins on BSM exhibited superior lubrication properties, followed by lupine protein. BSM acted synergistically with plant proteins forming a compact film (Fig. S4d, f, h‡), carrying the attributes of both components, creating a film that had superior lubricating properties as compared to BSM or plant proteins alone. Furthermore, as was shown in the past, the small molecular weight proteins could act as molecular glue with BSM, aiding towards the formation of a more compact layer, which in turn enhances the lubricating properties of the BSM-plant protein film.^25^

At high loads at pH 7.0 and 10 mM NaCl, all systems exhibit higher *μ*_eff_ due to dehydration and/or protein removal. Lupine protein exhibits the highest *μ*_eff_, due to its low surface coverage and low affinity towards the PDMS surface. Pea exhibits a lower friction coefficient than lupine, due to increased affinity and surface coverage. Interestingly, although potato protein exhibits the lowest affinity and similar levels of hydration as compared to lupine and pea, it exhibits similar *μ*_eff_ with pea. However, it appears that the affinity measured from the initial rate of adsorption can be misleading when comparing proteins with different sizes of aggregates and degrees of polydispersity (Table S1‡). Therefore, in the case of potato protein, the lower *μ*_eff_ could be explained by the adsorption of smaller molecules present in potato specifically, that have high affinity and are able to remain on the PDMS surface during sliding and, thus, aid lubrication. BSM improves lubrication in lupine and pea by combining the increased affinity of plant proteins with the superior hydration of BSM to form a protein layer with superior lubricating properties. However, *μ*_eff_ is increased when BSM is combined with potato. As explained earlier, this could be caused by the lower affinity of potato towards PDMS as well as unfavourable interactions between the two proteins.

## 3. Conclusion

**Fig. 8:**
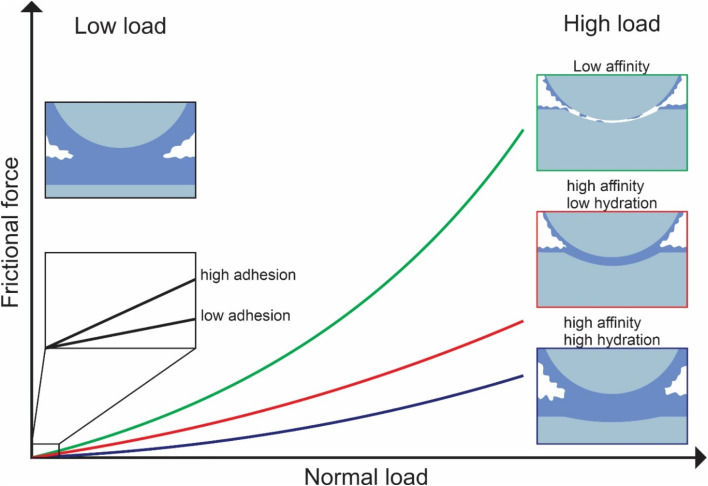
Schematic representation of the factors affecting lubrication at nanoscale.

In this study, we investigated the effect of three common types of plant proteins on friction under varying loads, revealing a complex yet explicable interplay between (a) adhesion between sliding bodies in the presence of protein, (b) protein affinity towards the surface which affects resistance to removal by wear, and (c) degree of hydration affecting overall frictional properties, which in turn are modified by pH and ionic strength (schematic is shown in Fig. 8). The combination of FFM with the soft contact surfaces employed in this study (*E* ∼150 kPa), has brought the tribological measurements for the first time to biologically realistic contact pressures (<50 kPa) particularly in the lower load conditions (at <10 nN normal force), unachieved by any macroscopic experiments to date.

We reveal that at first, protein affinity towards a given substrate is essential to ensure good lubricating properties of proteins. Having achieved sufficient adsorption, the degree of hydration of the adsorbed film becomes crucial since it reduces friction. Besides these two factors, surface interactions between the two protein-coated surfaces chiefly govern the lubricating performance of plant proteins at low loads since the adhesive forces between bodies are comparable to the observed friction force. It is also possible that at low loads surface roughness is another factor affecting friction, with increased roughness caused by the plant protein aggregates having increased friction, however, further investigation is needed to investigate roughness effects.

Although proteins with high affinity can have good lubricating properties even if the film is poorly hydrated, the opposite is not true; a highly hydrated film may not have superior lubricating properties unless the affinity towards the surface is not sufficient to maintain the film on the surface upon sliding. This was highlighted in the case of BSM where a highly hydrated film with poor affinity had very poor lubricating properties. However, upon combining BSM with plant proteins, which is much more representative of physiological conditions where the inner epithelium is coated with a mucosal layer, the resulting plant protein-BSM film has superior lubricating properties since it combines the high affinity of the plant proteins with the high hydration of BSM. However, at higher loads, many, if not most, plant proteins experienced a higher friction coefficient due to dehydration and protein removal caused by sliding. Generally, an increase in ionic strength increases hydration since protein can hold more water molecules due to an increased number of ions, while it also increases affinity towards the surface. As a result, the protein film becomes more resistant to dehydration and hinders protein removal caused by sliding. However, it could also lead to the formation of larger aggregates, which are jamming the contact thereby increasing friction coefficient.

In particular, among the tested proteins, pea protein seems to stand out in its lubrication performance as compared to lupine and potato, driven by an increased affinity towards the hydrophobic PDMS surface and a more hydrated protein film that aids lubrication. Although the trend is similar to what has been observed previously in macroscopic frictional response particularly at low protein concentrations as used in the current study, the differences in frictional behaviour of plant protein types are far more significant at the nanoscale owing to single papillae-level resolution and clear molecular mechanism behind such behaviour is laid out for the first time. The current work offers novel insights into the nanotribological performance of plant proteins pinpointing the role of adhesion, affinity and protein hydration as well as load dependency, that can be tuned either by plant protein type or subtle manipulation of environmental factors (pH, ionic strength) and holds great potential for future development of sustainable food and biomaterials where optimum lubrication is a key necessity.

## 4. Experimental section

### 4.1. Materials

Polydimethylsiloxane (PDMS) (Sylgard®184 and Sylgard®527) was purchased from Farnell, UK. HEPES (4-(2hydroxyethyl)-1-piperazineethanesulfonic acid), sodium dodecyl sulphate (SDS), ammonia (NH_3_) solution (25%), sodium chloride (NaCl), Hellmanex™ III cleaning solution, ethanol absolute, isopropanol, and toluene were purchased from Fisher Scientific, UK. Araldite 2-part epoxy adhesive was purchased from RS Components Ltd, UK. Sulphuric acid (H_2_SO_4_) was purchased from VWR International Ltd, UK. Hydrogen peroxide (H_2_O_2_) solution (30% wt%) was purchased from Sigma-Aldrich Company Ltd, UK. Silicon wafers were purchased from Agar Scientific Ltd, UK. Silicon-coated quartz crystal microbalance with dissipation monitoring (QCM-D) sensors (QSX-303, 5 MHz) were purchased from Biolin Scientific, UK. Atomic force microscopy (AFM) cantilevers (HQ:CSC37/tipless/Cr–Au) were purchased from Windsor Scientific Ltd, UK. Pea protein concentrate (Nutralys S85 XF) containing 85% protein was kindly gifted by Roquette (Lestrem, France). Potato protein isolate was purchased from Guzman Gastronomía (Barcelona, Spain) containing 91% protein. Lupine protein isolate containing 91% protein was purchased from Prolupin GmbH (Grimmen, Germany). Milli-Q water (resistivity of 18 MΩ cm by Milli-Q apparatus, Millipore Corp., USA) was used for the preparation of buffer. Protein solutions (1 mg mL^−1^) were prepared in 10 mM HEPES buffer in presence of 10 or 50 mM NaCl, adjusted to pH 3.0 or 7.0. The aforementioned protein were a mixture of several proteins and thus were highly polydisperse in nature showing various degree of aggregation with mean hydrodynamic diameters (*d*_H_) ranging from 25 nm to 244 nm.^27^ The proteins were negatively charged at neutral pH (see Table S1‡ for the physicochemical characteristics).

### 4.2. PDMS substrate preparation

Thin and ultrathin films of PDMS were prepared on silicon wafers and silicon-coated QCM-D crystals, respectively. For the preparation of PDMS-coated silicon substrates, Sylgard®184 (10 : 1 w/w base to curing agent) and Sylgard®527 (1 : 1 w/w Part A to Part B) were mixed using a planetary mixer (Thinky ARE-250, Intertronics, UK) for 60 s at 2000 rpm, followed by degassing for 90 s at 2200 rpm. Subsequently, the above products were mixed (Sylgard®184 : Sylgard®527) in 9 : 91 (9%) ratio, using the planetary mixer for 60 s at 2000 rpm followed by degassing for 90 s at 2200 rpm, to create PDMS substrates with elastic modulus of 150 kPa based on a previously described method.^18^ A droplet of 100 μL of the above elastomer was placed on a static silicon substrate and was rotated for 30 s at 4000 rpm with an acceleration of 2000 rpm s^−1^, using a spin-coater (Laurell technologies corporation, USA), to prepare film with a thickness of approximately 20 μm.^47^ Subsequently, the PDMS-coated substrate was placed on a hot plate at 80 °C for 30 min, followed by curing in a vacuum oven at 80 °C for 24 h. Lastly, the prepared PDMS-coated substrates were immersed in toluene for 24 h to remove the uncured PDMS, followed by 12 h storage in the vacuum oven at 80 °C to remove the toluene. For the preparation of the PDMS-coated QCM-D sensors, Sylgard®184 (10 : 1 w/w base to curing agent) was dissolved in toluene to prepare a 0.5 wt% solution and was stirred for 24 h. Subsequently, a 100 μL droplet of the PDMS solution was placed on a static silicon-coated quartz crystal and was rotated for 30 s at 5000 rpm with an acceleration of 2500 rpm s^−1^, using a spin-coater, to prepare PDMS films of approximately 10 nm thickness.^48^ The PDMS-coated QCM-D crystals were then placed on a hot plate at 80 °C for 30 min, followed by curing in a vacuum oven at 80 °C for 24 h. Prior to use, all PDMS-coated substrates were cleaned by immersion in toluene for 30 s, followed by immersion for 30 s in isopropanol, then MilliQ water for 5 min, followed by drying with pure nitrogen gas, and allowing any remaining solvent to evaporate for at least 1 h in an open container in a fume hood.

### 4.3. Fabrication of PDMS microspheres

PDMS Sylgard®184 (10 : 1 w/w base to curing agent) was mixed thoroughly using a planetary mixer for 60 s at 2000 rpm, followed by degassing for 90 s at 2200 rpm. Subsequently, 0.6 g of the above mixture were added to 30 mL of 15 wt% polyvinyl alcohol (PVA) aqueous solution to create PDMS-in-PVA emulsion droplets, and was stirred for 12 h at room temperature, followed by 12 h stirring at 80 °C. The resulting PDMS microparticles (elastic modulus 2.0 MPa)^18^ were centrifuged at 4000 rpm and washed with MilliQ water several times to ensure removal of any residual PVA.^49^

### 4.4. Fabrication of AFM colloidal probes

Rectangular tipless cantilevers with a spring constant ranging between 0.3 and 0.8 N m^−1^ were used for the fabrication of colloidal probes. Initially, the normal spring constant of the tipless cantilevers was determined using thermal tuning provided by Nanoscope software v9. Subsequently, spherical PDMS colloidal particles (diameter ≈ 10 μm) were attached to the end of the cantilevers using 2-part epoxy glue.

### 4.5. Scanning electron microscopy

An EVO MA15 scanning electron microscope (Carl Zeiss, Jena Germany) was used to image the colloidal probe cantilevers, in order to acquire the precise cantilever's dimensions, as well as the diameter of the attached particles.

### 4.6. Atomic force microscopy (AFM)

All AFM measurements were acquired using a Multimode 8 AFM (Bruker, USA) equipped with a Bruker Nanoscope V controller. The normal sensitivity and the spring constant were calibrated as detailed by Hutter and Bechhoefe.^50^ The lateral spring constant and the lateral sensitivity of the cantilevers were calculated by beam mechanics,^51^ using the cantilever and particle dimensions acquired with SEM. The AFM experiments were performed in a liquid cell loaded with the appropriate buffer. The samples were prepared as followed; approximately 200 μL of the desired protein solution was deposited on the PDMS sample and was let to adsorb for an hour. Subsequently, to remove non-adsorbed protein molecules that could adsorb on the cantilever and interfere with the laser signal, the protein solution was exchanged with buffer, ensuring that the sample always remained hydrated. Finally, the samples were transferred to the AFM for measurements.

### 4.7. AFM imaging

Topographic images of the uncoated, as well as protein-coated surfaces, were acquired by PeakForce Tapping™ (PFT) imaging technique, which reduced damage arising from lateral forces. Imaging was performed at room temperature in a liquid environment using a fused silica liquid cell loaded with buffer solution and ScanAsyst-Fluid + cantilevers (Bruker, USA). The PeakForce setpoint was set at 50 pN, the tapping frequency was 4 kHz, while the scanning rate was 1 Hz. Images were acquired with 512-pixel resolution and were subjected to 1st order flattening to remove tilt and offset of each line.

### 4.8. Friction force microscopy (FFM)

Frictional forces were calculated from friction loops, by recording the lateral voltage signal of the cantilever as the cantilever slides over the PDMS substrate, which are equal to half the difference between the average lateral voltage values obtained during the forward and reverse scan direction.^52,53^ The raw signal, acquired by Nanoscope, was processed with custom scripts developed in MATLAB (MathWorks). Experiments were performed using PDMS probes in the presence of plant protein solutions. Although tongue surface has a tendency to be hydrophobic and is weakly polar (60–70°),^19,54^ the static contact angle is far from that of PDMS (110°),^26,46^ latter is highly hydrophobic. However, PDMS is conventionally used in literature as the chosen substrate to understand frictional behaviour of proteins and therefore was used in this study for comparison. In order to be close to real tongue surface in terms of wettability, BSM was used to primarily coat the PDMS surfaces, BSM here acted as an approximate for salivary coating.^25,33^ The friction was measured starting from 0 nN normal load and increasing progressively to 90 nN, using a sliding speed of 5 μm s^−1^, over a scan size of 5 μm. A distance of 500 nm was kept between consecutive scan lines to ensure that the colloidal probe was sliding on a fresh surface every time and ensure steady conditions, while each scan was taken on a new area.

### 4.9. Quartz crystal microbalance with dissipation monitoring (QCM-D)

A quartz crystal microbalance with dissipation monitoring (QCM-D, E4 system, Q-sense, Sweden) was used to measure real-time adsorption of lupine, pea, and potato protein on PDMS-coated surfaces. A peristaltic pump (Ismatec, Germany) was used to inject the buffer at the right pH and protein solutions into the QCM-D chamber, using a flow of 100 μL min^−1^ at 25 °C. Initially, buffer solution was injected into the QCM-D chamber until a stable frequency and dissipation baseline was achieved. For the adsorption on bare PDMS surfaces, the protein solution was injected into the system and was left to adsorb, allowing the system to equilibrate, followed by rinsing with buffer to remove loosely bound protein molecules for at least 30 min. For adsorption onto the BSM-coated PDMS sensors, BSM was first injected into the system and left to adsorb until equilibration, before rinsing with buffer for at least 30 min. Subsequently, protein solutions were injected and let to adsorb on the BSM film, before finally rinsing with buffer. All sensors were used only once to ensure the cleanliness of the PDMS surfaces. Frequency and dissipation data were collected by Qsoft software (Q-Sense, Sweden) and were analysed by Dfind (Q-Sense, Sweden). Subsequently, the 3rd to 11th overtones were fitted using a Voigt “Smartfit” model for viscoelastic films provided by Dfind to obtain the film thickness.^36^ Each sample was measured in triplicates and means and standard deviations were reported.

### 4.10. Statistical analysis

Significant differences between samples were determined using one-way ANOVA with *post hoc* Tukey's multiple comparison test using SPSS software (IBM, SPSS statistics) and 95% level of confidence.

## Conflicts of interest

The authors declare no conflict of interest.

## Supplementary Material

NA-005-D2NA00696K-s001
