# Supplementary material for: Frictional behaviour of plant proteins in soft contacts: unveiling nanoscale mechanisms
Source: Nanoscale Adv. 2022 Dec 26;5(4):1102–14. doi: 10.1039/d2na00696k (PMC9926882; doi:10.1039/d2na00696k)
Supplement: NA-005-D2NA00696K-s001 [file NA-005-D2NA00696K-s001.pdf]

Electronic Supplementary Material (ESI) for Nanoscale Advances.
This journal is © The Royal Society of Chemistry 2022

Supporting Information

Frictional behaviour of plant proteins in soft contacts: unveiling nanoscale mechanisms

Evangelos Liamas[1†], Simon D. Connell[2], Anwesha Sarkar[1*]

[1]Food Colloids and Bioprocessing Group, School of Food Science and Nutrition, University of Leeds, Leeds, LS2 9JT, UK

[2] Molecular and Nanoscale Physics Group, School of Physics and Astronomy, University of Leeds, Leeds, LS2 9JT, UK

†Present address: Unilever Research & Development Port Sunlight, Quarry Road East, Bebington Merseyside CH63 3JW, UK

Corresponding Author e-mail: A.Sarkar@leeds.ac.uk

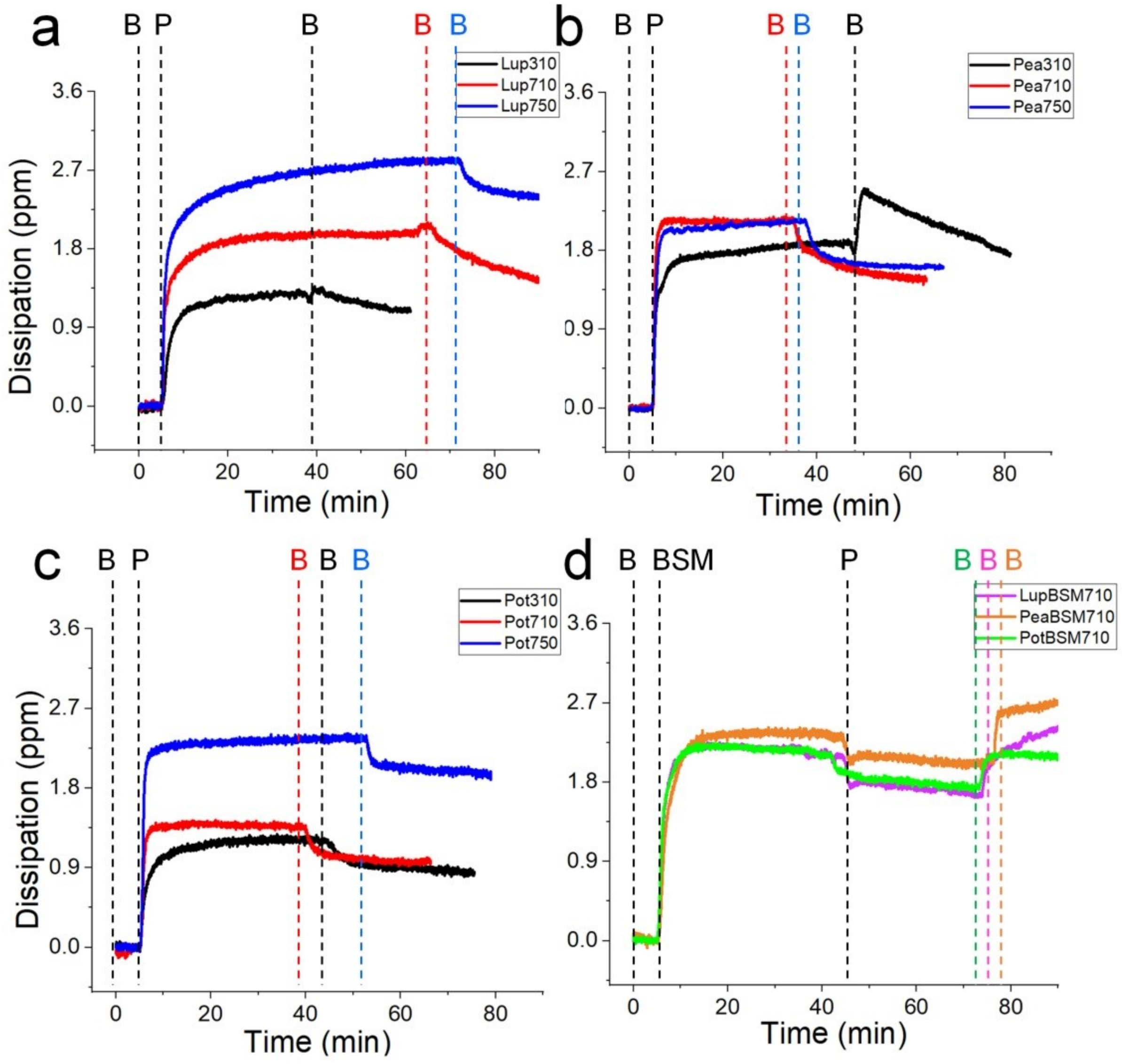

Figure S1. Mean dissipation (5th overtone) of lupine (a), pea (b) and potato (c) protein films (n=3) adsorbed on bare polydimethylsiloxane (PDMS) coated sensors, acquired by quartz crystal microbalance with dissipation monitoring (QCM-D). At bare PDMS surfaces measurements were taken in presence of 1 mg mL^{-1} protein dissolved at three different pH and ionic concentration combinations: 10 mM NaCl at pH 3.0 (Lup310, Pea310, Pot310), 10 mM NaCl at pH 7.0 (Lup710, Pea710, Pot710), and 50 mM NaCl at pH 7.0 (Lup750, Pea750, Pot750). Mean dissipation (5th overtone) of lupine, pea and potato protein films (n=3) adsorbed on (d) BSM-coated surfaces were performed in presence of 1 mg mL^{-1} protein dissolved in 10 mM NaCl at pH 7.0 (LupBSM710, PeaBSM710, PotBSM710). In the plots, steps B, P, and BSM refer to buffer rising, protein addition, and mucin coating, respectively.

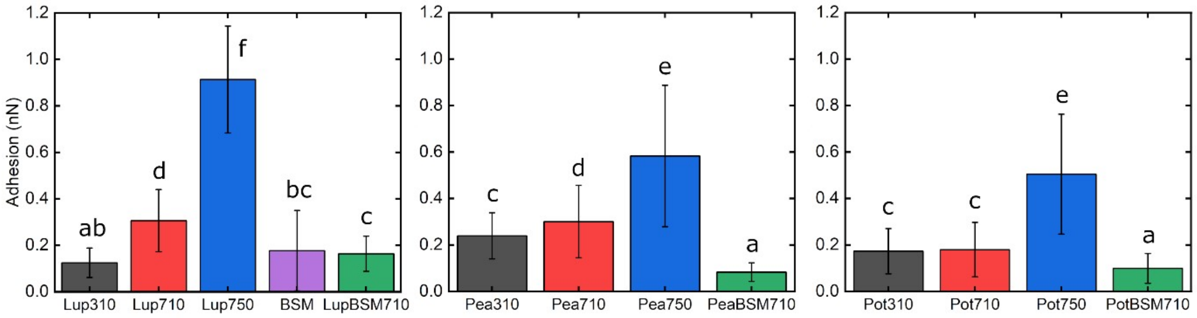

Figure S2. Adhesion between a polydimethylsiloxane (PDMS) colloidal probe (E=2 MPa) against bare and mucin-coated PDMS substrates (E=150 kPa), in presence of lupine, pea, and potato protein solutions, acquired by AFM force spectroscopy. Using bare PDMS surfaces, measurements were taken in the presence of 1 mg mL^{-1} protein dissolved at three different pH and ionic concentration combinations: 10 mM NaCl at pH 3.0 (Lup310, Pea310, Pot310), 10 mM NaCl at pH 7.0 (Lup710, Pea710, Pot710), and 50 mM NaCl at pH 7.0 (Lup750, Pea750, Pot750). Measurements on BSM-coated surfaces were performed in the presence of 1 mg mL^{-1} plant protein dispersions dissolved in 10 mM NaCl at pH 7.0 (Lup/BSM710, Pea/BSM710, Pot/BSM710). Samples with the same letter do not differ significantly (*p>0.05*) according to Tukey's test.

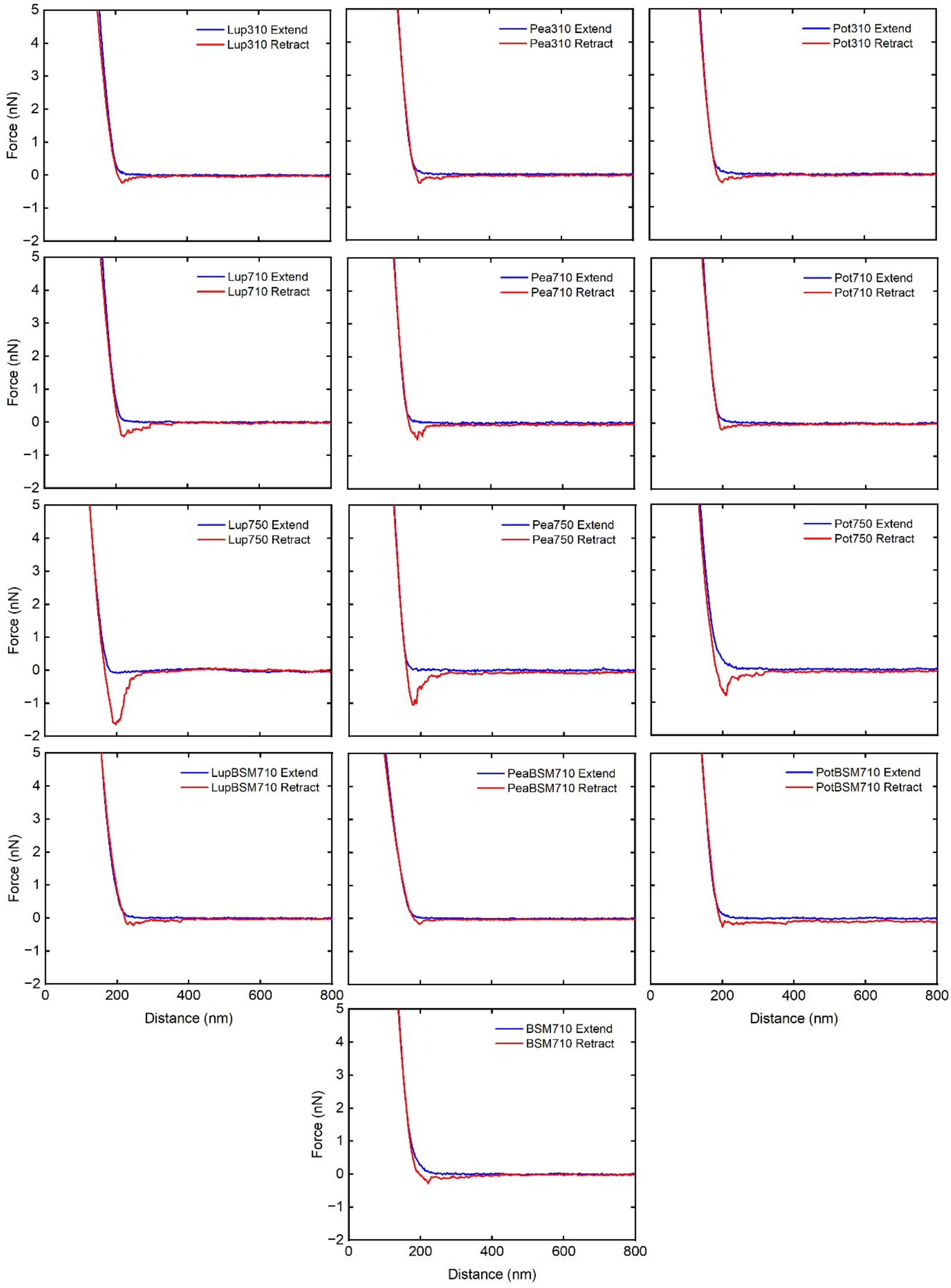

Figure S3. Characteristic force distance (F-D) curves between a between a polydimethylsiloxane (PDMS) colloidal probe (E=2 MPa) against bare and mucin-coated PDMS substrates (E=150 kPa), in presence of lupine, pea, and potato protein solutions, acquired by AFM force spectroscopy.

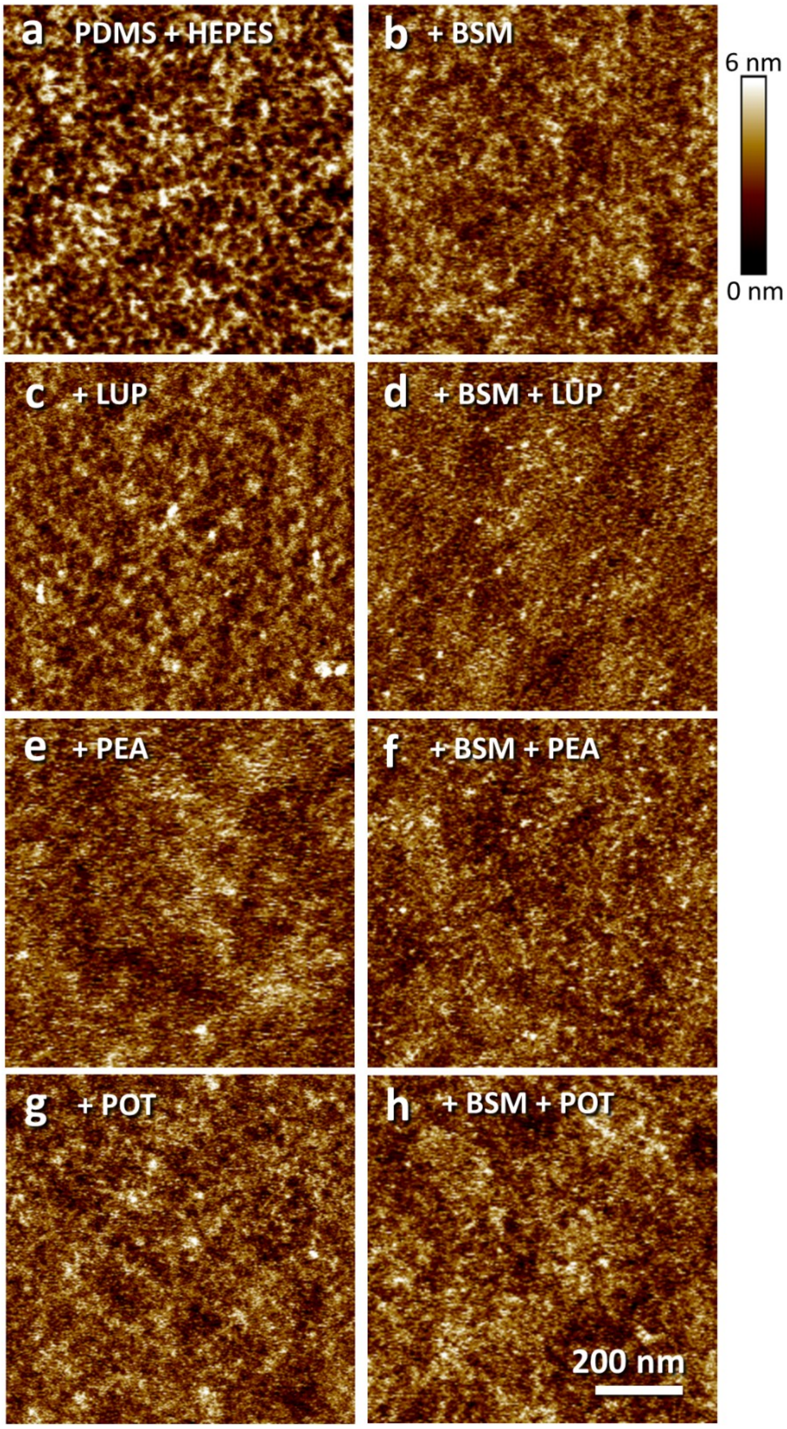

Figure S4. Surface morphology of PDMS substrates coated with proteins. Topographic peak force tapping mode AFM images in HEPES buffer of PDMS substrates without any protein (a) or coated with BSM (b), lupine protein (c), BSM+lupine protein (d), pea protein (e), BSM+pea protein (f), potato protein (g), and BSM+potato protein (h) at pH 7.0 and 10 mM NaCl.

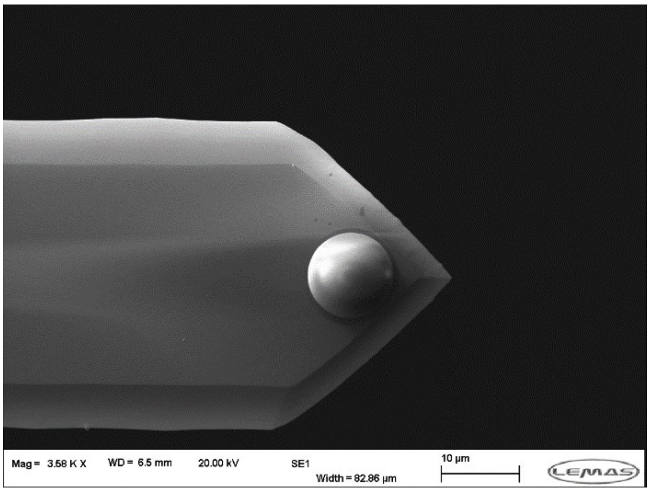

Figure S5. Polydimethylsiloxane (PDMS) colloidal probe as acquired by scanning electron microscopy.

Table S1. Protein characteristics.

Protein sample	Nomenclature	Isoelectric point (pI)	Hydrodynamic diameter (d_H) (nm)	Polydispersity index (PDI)	ζ-potential (mV)
Lupine	Lup710	4.0 [1]	116 [2]	0.3 [2]	-23.0 [2]
Pea	Pea710	4.0 [3]	244 [2]	0.4 [2]	-20.6 [2]
Potato	Pot710	4.5 – 5.0 [4]	25 [2]	0.7 [2]	-22.4 [2]

Table S2. RMS roughness and Z range of bare PDMS and protein-coated PDMS surfaces as acquired by atomic force microscopy.

Solution	RMS (R_q) roughness (nm)	Z range or peak-to-peak (nm)
HEPES	1.24	25.0
Lup710	0.98	17.5
Pea710	0.90	11.4
Potato710	0.93	13.7
BSM710	0.91	8.8
LupBSM710	0.82	11.9
PeaBSM710	0.84	10.1
PotBSM710	0.90	16.2

References

1. S. L. Rodríguez-Ambriz, A. L. Martínez-Ayala, F. Millán and G. Dávila-Ortíz, *Plant Foods for Human Nutrition*, 2005, **60**, 99-107.
2. B. Kew, M. Holmes, M. Stieger and A. Sarkar, *Food Hydrocolloids*, 2021, **116**, 106636.
3. E. Adal, A. Sadeghpour, S. Connell, M. Rappolt, E. Ibanoglu and A. Sarkar, *Biomacromolecules*, 2017, **18**, 625-635.
4. J. M. Schmidt, H. Damgaard, M. Greve-Poulsen, A. V. Sunds, L. B. Larsen and M. Hammershøj, *Food Hydrocolloids*, 2019, **96**, 246-258.
